# 

**Published:** 1874-05

**Authors:** 


### Table of Contents.

### ORIGINAL COMMUNICATIONS.

Sympathetic Ophthalmia.... *By A. W. Calhoun, M.D., Atlanta, Ga*... 65
Fœtal Deformities.... *By J. A. Meek, Jonesboro, Ark*.......... 71
Hydrate of Chloral.... *By E. M. Nolan, M.D., McDonough, Ga*...... 73
Maggots in Externa Ear..... *By J. A. Meek, Jonesboro, Ark*.......... 74
Saccharated Calomel........ *By "B.," Louisville, Ky*. ........... 75

### MEDICAL SOCIETIES AND CLINICS.

Atlanta Academy of Medicine........................ 76
Clinic Atlanta Medical College........................ 81

### OUR STATE SOCIETIES.

Medical Association of Georgia........................ 84
Alabama State Medical Association........................ 88
Tennessee State Medical Society........................ 90
Kentucky State Medical Society........................ 92

### OUR EXCHANGES.

A Cremation Elegy (Poetical)........................ 95
Modern Thought (Poetical)........................ 96
Puerperal Convulsions—treatment........................ 97
Gastro-Enteritis—Delirium tremens........................
Granular-Lids—Abscesses........................ 99
Spermatorrhœa—Delirium tremens........................
Intermittent fever........................ 100
Eczema capitis—Coryza........................ 101
Hæmatocele, perinterine........................ 102
Method of Surgical Diagnosis Erichsen........................ 103
New local anæsthetic—Bromide Calcium........................ 105
"Dr. Johannes A. Jones"........................ 106
Post-partum hemorrhage—Delirium-tremens........................
Intra-uterine injections—Hydrothorax........................ 107
Local treatment pulmonary cavities by injection........................ 108
Calomel in Hepatic diseases........................ 110
Human rumination........................ 113
Pepsin........................
Poisoning by pseudo-homœopathy........................
Excision of the larynx........................ 115
Induction of labor in uræmia........................ 116

### BIBLIOGRAPHICAL.

Wood's Therapeutics and Mat. Med........................ 117
Reese's Toxicology........................ 119
Toner's Dictionary of Elevations........................ 120
Arthius on Static Electricity........................
Report of U. S. Marine Hospital........................
Pamphlets received........................ 121
Transactions Medical Society of Virginia........................ 122
Acknowledgments........................ 123

### EDITORIAL.

Cremation........................ 124
Meeting of Confederate Surgeons........................
Laws regulating the practice of medicine........................ 126
Georgia Medical Asssociation........................ 128

